# Energy-Efficient Optimal Power Allocation in Integrated Wireless Sensor and Cognitive Satellite Terrestrial Networks

**DOI:** 10.3390/s17092025

**Published:** 2017-09-04

**Authors:** Shengchao Shi, Guangxia Li, Kang An, Bin Gao, Gan Zheng

**Affiliations:** 1 College of Communications Engineering, PLA University of Science and Technology, No. 2 Biaoying, Qinhuai District, Nanjing 210007, China; shishengchao88@gmail.com (S.S.); ankang@nuaa.edu.cn (K.A.); feimaxiao123@gmail.com (B.G.); 2 Wolfson School of Mechanical, Electrical and Manufacturing Engineering, Loughborough University, Loughborough LE11 3TU, UK; g.zheng@lboro.ac.uk

**Keywords:** wireless sensor network, cognitive satellite, power allocation, energy efficiency, fading channels, interference power constraint, transmit power constraint

## Abstract

This paper proposes novel satellite-based wireless sensor networks (WSNs), which integrate the WSN with the cognitive satellite terrestrial network. Having the ability to provide seamless network access and alleviate the spectrum scarcity, cognitive satellite terrestrial networks are considered as a promising candidate for future wireless networks with emerging requirements of ubiquitous broadband applications and increasing demand for spectral resources. With the emerging environmental and energy cost concerns in communication systems, explicit concerns on energy efficient resource allocation in satellite networks have also recently received considerable attention. In this regard, this paper proposes energy-efficient optimal power allocation schemes in the cognitive satellite terrestrial networks for non-real-time and real-time applications, respectively, which maximize the energy efficiency (EE) of the cognitive satellite user while guaranteeing the interference at the primary terrestrial user below an acceptable level. Specifically, average interference power (AIP) constraint is employed to protect the communication quality of the primary terrestrial user while average transmit power (ATP) or peak transmit power (PTP) constraint is adopted to regulate the transmit power of the satellite user. Since the energy-efficient power allocation optimization problem belongs to the nonlinear concave fractional programming problem, we solve it by combining Dinkelbach’s method with Lagrange duality method. Simulation results demonstrate that the fading severity of the terrestrial interference link is favorable to the satellite user who can achieve EE gain under the ATP constraint comparing to the PTP constraint.

## 1. Introduction

The advancement in wireless communications and electronics has enabled the development of low-cost wireless sensor networks (WSNs), which have been widely used in various areas, such as monitoring, disaster relief and target tracking [1]. Since the sensing information must be transmitted to the remote monitoring hosts, the fundamental communication problems are important to WSNs [2]. However, the related researches have mainly focused on the terrestrial WSNs, which may be challenged by the operating environment, such as forest, wilderness and military environments [3,4]. With the obvious superiority in providing large coverage areas at low cost and supporting fixed and mobile services with various connecting modes, satellite systems have been widely utilized for wireless communications services to worldwide users, especially in the remote and underpopulated areas where terrestrial networks are economically and/or operationally infeasible [5,6]. Therefore, satellite-based sensor networks have drawn considerable attention and been investigated for various application scenarios [7,8,9].

Meanwhile, spectrum scarcity of the satellite communications is an urgent issue due to the increasing demand for the broadband applications and multimedia services. To alleviate pressure on limited spectral resources, cognitive radio (CR) as a promising technology to improve the spectrum efficiency (SE), has been introduced for satellite communications. In such a network, cognitive techniques can be applied in two satellite networks, or in satellite and terrestrials within the same frequency band [10,11,12].

Due to the easy implementation and high SE, the underlay technique is widely employed in CR networks, where the secondary user (SU) could simultaneously coexist with the primary user (PU) in the same band [13]. The premise is that the interference generated by the SU would not degrade the PU’s communication quality. Therefore, when the terrestrial system operates as the primary network and the satellite system serves as the secondary network [14], it is of crucial importance to design the efficient power allocation schemes for the satellite user in the uplink case. In this regard, the power allocation scheme is proposed for the fixed satellite services system in [15], where the primary system is fixed-service terrestrial microwave system. However, this scheme cannot be adopted into the fading channels. Considering the fading channel scenarios, optimal power control schemes are presented for non-real-time and real-time applications in [16,17], respectively, where the terrestrial cellular system operates as the primary system. The ergodic capacity of the satellite user is maximized in [16], which is an appropriate performance metric for non-real-time applications. In [17], delay-limited capacity and outage capacity are optimized for the real-time applications from the long-term and short-term perspectives, respectively. However, all the above-mentioned works aim to maximize the capacity of the satellite user and not consider the energy efficiency of the satellite user, which is the main objective in green cognitive radio networks.

According to the reports in [18,19], 2% to 10% of global energy consumption and 2% of the greenhouse gas are generated by information and communication technologies. Thus, in the cognitive radio networks, it is crucial to design the energy efficient transmission. The improved energy efficiency is a basic premise for secondary users to achieve high utilization of the limited transmit power which is consumed not only to improve spectrum efficiency but also implement some additionally important functionalities, e.g., spectrum sensing and reduce operational expenditure and the greenhouse effect. With the emerging environmental and energy cost concerns in communication systems, energy efficiency (EE) has become vital and inevitable in future satellite networks from both financial and ecological viewpoints [20,21]. Thus, the maximization of the EE instead of the capacity of the satellite is the novelty in this paper. The issue of optimal energy allocation and admission control is addressed for communications satellites in earth orbit in [22]. The authors in [21] make an overview of EE and satellite networking from a holistic perspective as well as the prospective greener architectures. The energy efficient power allocation problems in multibeam downlink satellite network is analyzed in [23]. Besides, the authors in [24] investigate the relationship between SE and EE for hybrid satellite terrestrial network, where overhead costs, transmission and circuit power, backhaul of gateway (GW), and density of small cells are taken into consideration. The energy efficiency of a multibeam downlink system is investigated in [25], which maximizes the ratio of system throughput over consumed power. However, to the best knowledge of the authors, energy-efficient power allocation problem in cognitive satellite terrestrial networks has not yet been solved in existing literature.

In this paper, a novel integrated wireless sensor and cognitive satellite terrestrial network architecture is first presented, where the cognitive satellite user plays the role of the sink for the terrestrial sensor network and the sensing data is transmitted through the satellite communication networks. Then, energy-efficient optimal power allocation schemes are proposed for non-real-time and real-time applications in cognitive satellite terrestrial networks, which aim to maximize the EE of the cognitive satellite user while guaranteeing the interference at the primary terrestrial user below an acceptable level. To guarantee the quality of the primary terrestrial user, average interference power (AIP) constraint is considered in the proposed schemes. To solve the nonlinear concave fractional programming problem, we combine Dinkelbach’s method [26] with Lagrange duality method [27] and decouple the problem into multiple parallel subproblems. Then, an iterative algorithm is presented to search the optimal transmit power of the satellite user. Extensive numerical results evaluate the performance of the proposed energy efficient power allocation schemes and show that the fading of the terrestrial interference link is favorable to the satellite user who can achieve EE gain under the ATP constraint comparing to the PTP constraints.

The remainder of this paper is structured as follows: Section 2 presents the system model and link budget. The energy-efficient optimal power allocation problem is formulated for both non-real-time and real-time applications and the solutions are derived in Section 3. Section 4 presents simulation results. We conclude this paper in Section 5.

## 2. System Model

**Figure 1:**
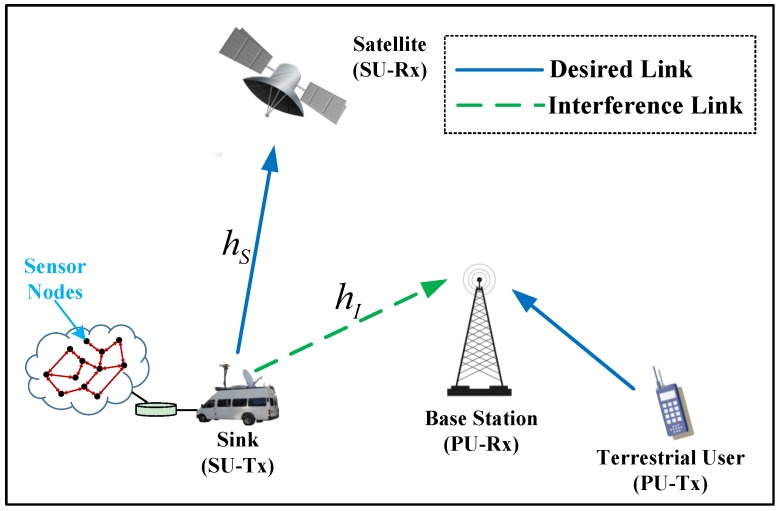
The architecture of the integrated wireless sensor and cognitive satellite terrestrial networks.

Figure 1 shows the architecture of the integrated wireless sensor and cognitive satellite terrestrial networks, where the mobile satellite terminal plays the role of the sink for the terrestrial sensor network. In this system, an uplink cognitive satellite terrestrial network consisting of one primary terrestrial network and one secondary satellite network is considered, where the satellite system shares the spectral resource with terrestrial system to improve the spectral efficiency. In the considered architecture, the satellite network (e.g., DVB-SH) acts as the secondary system, whereas the terrestrial cellular network (e.g., UMTS or LTE) corresponds to the primary system [16,17]. Herein, we focus on the underlay scenario as mentioned above. In addition, the weak interference from primary terrestrial user to the satellite can be negligible due to the large distance [28].

In traditional WSNs, sensor nodes are distributed in the sensing field whereupon detecting some events of interest, nodes report the sensed event back to some static sink(s) through multi-hop or single hop communication. One major drawback of such communication infrastructures is that the sensor nodes close to the sink will consume more energy, and thus their energy supply will be rapidly depleted [29]. To deal with this issue, the concept of mobile sink was introduced in [30,31], that not only results in balanced energy consumption among the nodes but can also be exploited to connect isolated segments of the network [32]. Moreover, some applications explicitly require sink mobility in the sensor field. For instance, a rescuer equipped with a PDA moves around in a disaster area to search any survivors [33], and a farmer while walking around a field would be interested in knowing which segment of the field requires watering, fertilizers, etc. Thus, the sink in this paper i.e., the satellite user is selected as a mobile terminal.

The operating power refers to the power needed for running the network equipment, e.g., the satellite terminal. In the considered system model, the satellite terminal is a vehicle equipment, which is commonly powered by on-board batteries, that is to say, the satellite terminal is limited in energy storage capacity. In this regard, energy efficiency is a fundamental constraint in the operation and design of communication networks consisting of battery-operated terminals. In addition, DVB-SH transmissions are subject to long-fading durations which degrade the quality of experience if not tackled efficiently. The long propagation delay in satellite networks (especially in GEO-based networks) and fast changing link conditions impose challenges on the energy efficiency optimizations [21]. Therefore, it is of importance to optimize the power allocation mechanism from the energy efficiency perspective of the satellite vehicle terminal.

When the transmit power of the satellite user is $P_{t}$, the receive power $P_{r}$ at the satellite can be calculated as

(1) $$P_{r}=P_{t}G_{t}\left(\theta \right)G_{r}\left(\varphi \right)L_{S}h_{S},$$

where $G_{t}\left(\theta \right)$ is the transmit antenna gain of the satellite user, $G_{r}\left(\varphi \right)$ denotes the receive antenna gain at the satellite, which can be obtained as

(2) $$G_{t}\left(\theta \right)=\left\{\begin{array}{cc} G_{t,\max}, & 0^{\circ }<\theta <1^{\circ }\\ 32-25\log\theta , & 1^{\circ }<\theta <48^{\circ }\\ -10, & 48^{\circ }<\theta <180^{\circ } \end{array}\right.,$$

(3) $$G_{r}\left(\varphi \right)=G_{r,\max}{\left(\frac{J_{1}\left(u\right)}{2u}+36\frac{J_{3}\left(u\right)}{u^{3}}\right)}^{2},$$

where θ is the elevation angle, $G_{r,\max}$ is the maximum beam gain at the onboard antenna boresight and $J\left(\cdot \right)$ is the Bessel function. Moreover, $u=2.07123\frac{\sin\varphi }{\sin\varphi_{3\mathrm{dB}}}$, where φ is the angle between the location of the satellite user and the beam center with respect to the satellite, and $\varphi_{3\mathrm{dB}}$ is the 3-dB angle.

$L_{S}$ is the free space loss of the secondary link. Besides, $h_{S}$ is the fading channel power gain of the secondary link. Herein, we employ the widely-adopted Shadowed-Rician fading model with closed formula, which can be used for mobile/fixed terminals operating in various propagation environment. According to [34], the probability density function (PDF) of $h_{S}$ is shown as

(4) $$f_{h_{S}}\left(x\right)=\alpha \exp\left(-\beta x\right){}_{1}F_{1}\left(m_{S},1,\delta x\right),$$

where ${}_{1}F_{1}\left(\cdot ,\cdot ,\cdot \right)$ denotes the confluent hypergeometric function [35] and α, β and δ can be calculated as

(5) $$\begin{array}{c} \alpha =\frac{1}{2b_{S}}{\left(\frac{2b_{S}m_{S}}{2b_{S}m_{S}+\Omega_{S}}\right)}^{m_{S}},\\ \beta =\frac{1}{2b_{S}},\\ \delta =\frac{\Omega_{S}}{2b_{S}\left(2b_{S}m_{S}+\Omega_{S}\right)}, \end{array}$$

where $2b_{S}$ is the average power of the scatter component, $\Omega_{S}$ is the average power of the line-of-sight (LOS) component and $m_{S}$ is the Nakagami fading parameter.

Similarly, the interference power $P_{i}$ at the base station (BS) in primary terrestrial networks can be calculated as

(6) $$P_{i}=P_{t}G_{t}\left(\theta^{\prime }\right)G_{BS}L_{p}h_{I},$$

where $G_{t}\left(\theta^{\prime }\right)$ is the equivalent transmit antenna gain for terrestrial interference link with off-axis angle $\theta^{\prime }\;=\;\arccos\left(\cos\left(\theta \right)\cos\left(\psi \right)\right)$ and ψ denotes the angle between the over horizon projected main lobe of the satellite user and the BS [36]. In addition, $G_{BS}$ is the receive antenna gain at the BS and, $L_{p}$ and $h_{I}$ are free space loss and the fading channel power gain of the terrestrial interference link, respectively. As for $h_{I}$, Nakagami fading distribution is considered and $h_{I}$ follows the PDF given by [16]

(7) $$f_{h_{I}}\left(x\right)=\frac{\varepsilon^{m_{I}}x^{m_{I}-1}}{\Gamma \left(m_{I}\right)}\exp\left(-\varepsilon x\right),$$

where $\Gamma \left(\cdot \right)$ is the Gamma function [35], $m_{I}$ is the Nakagami fading parameter, $\Omega_{I}$ is the average power and $\varepsilon \;=\;m_{I}/\Omega_{I}$. For brevity, we denote $G_{S}=G_{t}\left(\theta \right)G_{r}\left(\varphi \right)L_{S}$ and $G_{I}=G_{t}\left(\theta^{\prime }\right)G_{BS}L_{p}$ in the rest of the paper.

To facilitate the analysis of the average EE limits in cognitive satellite terrestrial networks, it is assumed that the satellite user has perfect channel state information (CSI) about $h_{S}$ and $h_{I}$ at all fading states. Note that $h_{S}$ can be obtained by estimating it at the satellite and sending it back to the satellite user through a feedback link. Furthermore, $h_{I}$ can be obtained through cooperation with the BS, or from a third party such as the spectrum manager [37].

## 3. Energy-Efficient Optimal Power Allocation

Since the demand for global coverage providing broadband services is increasing, supporting interactive multimedia traffic is expected as an essential component in satellite systems. In addition, the satellite traffic could be divided into two classes: non-real-time applications, such as email, remote login or ftp and real-time applications, such as voice and video.

### 3.1. Energy-Efficient Optimal Power Allocation for Non-Real-Time Applications

In this section, we propose two energy-efficient optimal power allocation schemes for non-real-time applications. To regulate the transmit power limit of the satellite user, average transmit power (ATP) constraint and peak transmit power (PTP) constraint are adopted in the two schemes, respectively. From the perspective of guaranteeing the primary terrestrial user’s communication quality, it is necessary to impose interference power constraint on the satellite user. Compared with the peak interference power (PIP) constraint, the average interference power (AIP) constraint can not only protect PU better, but also provide higher capacity for SU [38]. Thus, we employ AIP constraint in both schemes herein.

#### 3.1.1. Average Transmit Power Constraint

Ergodic capacity (EC) is an appropriate performance metric for non-real-time applications, which can be obtained by averaging over all states of an ergodic fading channel. Therefore, EE for non-real-time applications can be denoted as the ratio of the EC to the average power consumption [37]. Employing the ATP constraint, EE maximization problem is formulated as

(8) $$\begin{array}{c} \max_{P_{t}}H\left(P_{t}\right)=\frac{E\left[\log_{2}\left(1+\frac{P_{r}}{N_{s}}\right)\right]}{E\left(\xi P_{t}+P_{c}\right)},\\ s.t.\left\{\begin{array}{c} E\left(P_{t}\right)\leq P_{av}\;\left(a1\right)\\ E\left(P_{i}\right)\leq I_{th}\;\;\;\;\left(b\right) \end{array}\right. \end{array}$$

where $N_{s}$ represents the noise power, ξ and $P_{c}$ are the amplifier coefficient and the constant circuit power consumption of the satellite user, respectively and $E\left(\cdot \right)$ denotes the statistical expectation. Moreover, $P_{av}$ and $I_{th}$ denote the ATP constraint limit and the AIP constraint limit, respectively.

It can be proved that (8) is a nonlinear concave fractional programming problem. Therefore, the following conclusion can be obtained.

**Theorem** **1.** Any local maximum in *(8)* is a global maximum and there is at most one maximum since *(8)* is strictly quasiconcave.

**Proof.**  Because the numerator of $H\left(P_{t}\right)$ is strictly concave, (8) is strictly quasiconcave. In addition, since the numerator and denominator of $H\left(P_{t}\right)$ are differentiable and the numerator is strictly concave, (8) is strictly pseudoconcave [27]. Based on these results, when $\frac{dH\left(P_{s}\right)}{dP_{s}}=0$, $H\left(P_{t}\right)\leq H\left(P_{s}\right)$ would hold at any $P_{t}$. Thus, $H\left(P_{s}\right)$ can be proved to be the global maximum. ☐

Because (8) is a nonlinear fractional program, according to Dinkelbach’s method [26], it can be equivalently formulated as the problem below with a parameter η

(9) $$T\left(\eta \right)=\max_{P_{t}\in S_{1}}E\left[\log_{2}\left(1+\frac{P_{r}}{N_{s}}\right)\right]-\eta E\left(\xi P_{t}+P_{c}\right),$$

where η is a non-negative parameter and $S_{1}$ denotes the set $S_{1}=\left\{P_{t}|P_{t}\in \left(a1\right)\cap \left(b\right)\right\}$. We can obtain the global maximum of (8) by solving (9). Furthermore, it is easy to prove that (9) is a convex problem. Thus, we can solve (9) by employing the Lagrange duality method since the duality gap is zero [27]. The Lagrangian function of (9) can be expressed as

(10) $$\begin{array}{c} L\left(P_{t},\tau ,\mu \right)=E\left[\log_{2}\left(1+\frac{P_{r}}{N_{s}}\right)\right]-\eta E\left(\xi P_{t}+P_{c}\right)-\tau \left[E\left(P_{t}\right)-P_{av}\right]-\mu \left[E\left(P_{i}\right)-I_{th}\right], \end{array}$$

where τ and μ are the non-negative Lagrangian multipliers related to (a1) and (b) in (8), respectively. Hence, the Lagrange dual function of (9) is given as

(11) $$g\left(\tau ,\mu \right)=\max_{P_{t}\geq 0}L\left(P_{t},\tau ,\mu \right).$$

Then, the dual problem of (9) can be presented as

(12) $$\min_{\tau ,\mu }g\left(\tau ,\mu \right).$$

Similar to [38], (12) can be decoupled into multiple parallel subproblems based on the Lagrange dual-decomposition method [27]. These subproblems have the same structure for each fading state. Therefore, given a particular fading state, the corresponding subproblem can be formulated as

(13) $$\max_{P_{t}\geq 0}D\left(P_{t}\right)=\log_{2}\left(1+\frac{P_{r}}{N_{s}}\right)-\eta \xi P_{t}-\tau P_{t}-\mu P_{i}.$$

We can obtain the global maximum of (9) by iteratively solving (13) for all fading states with the fixed τ and μ, and updating τ and μ by subgradient method [27]. Then, we can derive the optimal transmit power $P_{t}^{*}$ of (9) as shown in Theorem 2.

**Theorem** **2.** *The energy-efficient optimal transmit power for non-real-time applications with ATP constraint is given as*

(14) $$P_{t}^{*}={\left[\frac{1}{\left(\eta \xi +\tau +\mu G_{I}h_{I}\right)\ln2}-\frac{N_{s}}{G_{S}h_{S}}\right]}^{+},$$

*where ${\left[x\right]}^{+}=\max\left(0,x\right)$, which means the maximum between x and 0.*

We can see that (9) can be efficiently solved via (14) for a given η. To solve (8) and find the maximum EE $\eta^{*}$, we resort to the Dinkelbach’s method [26]. Then, we propose the iterative power allocation algorithm to solve (8), which is denoted by Algorithm 1. It has been proved that Dinkelbach’s method can converge to the optimal solution with a superlinear convergence rate [39,40]. The proof of the convergence is shown as below. Before the proof of convergence, two Lemmas are given as follows.

**Algorithm 1:** Iterative Power Allocation Algorithm for (8).  **Set parameters:**  $\xi_{0}>0$, $\xi_{1}>0$, $\xi_{2}>0$: Error tolerances;  $t_{1}>0$, $t_{2}>0$ : Step sizes;  $N_{i}$: Iteration number.  **Initialization:**  $\eta =\eta_{0}$, $\tau =\tau_{0}$, $\mu =\mu_{0}$, $\delta =\delta_{0}$;  Calculate $P_{t}^{0}$ using (14);  $\delta_{1}=\left|\tau^{k}\left(P_{av}-E\left({P_{t}}^{0}\right)\right)\right|$, $\delta_{2}=\left|\mu^{k}\left(I_{th}-E\left({P_{i}}^{0}\right)\right)\right|$.  **Search optimal values:**  n=0, $\eta_{0}=0$;  **While**
$\delta >\xi_{0}$  k=0;  Update τ and μ by subgradient method as follows:  **While**
$\delta_{1}>\xi_{1}$ or $\delta_{2}>\xi_{2}$   $\tau^{k+1}={\left[\tau^{k}-t_{1}\left(P_{av}-E\left({P_{t}}^{k}\right)\right)\right]}^{+}$;   $\mu^{k+1}={\left[\mu^{k}-t_{2}\left(I_{th}-E\left({P_{i}}^{k}\right)\right)\right]}^{+}$;   k=k+1;   Calculate $P_{t}^{k}$ using (14);   $\delta_{1}=\left|\tau^{k}\left(P_{av}-E\left({P_{t}}^{k}\right)\right)\right|$;   $\delta_{2}=\left|\mu^{k}\left(I_{th}-E\left({P_{i}}^{k}\right)\right)\right|$;  **End**;  $\eta_{n+1}=\frac{E\left[\log_{2}\left(1+\frac{{P_{r}}^{k}}{N_{s}}\right)\right]}{E\left(\xi {P_{t}}^{k}+P_{c}\right)}$;  $\delta =\eta_{n+1}-\eta_{n}$;  **End**;  $P_{t}^{*}=P_{t}^{k}$;  $\eta^{*}=\eta_{n+1}$.

**Lemma** **1.** *T(η) defined in* (9) *is strictly monotonic decreasing, i.e., $T\left(\eta^{♢}\right)<T\left(\eta^{♡}\right)$ if $\eta^{♢}>\eta^{♡}$.*

**Proof.**  Let $P_{t}^{♢}$ maximize $T(\eta^{♢})$, then

(15) $$\begin{array}{cc} T(\eta^{♢}) & =\max_{P_{t}}\left\{E\left[\log_{2}\left(1+\frac{P_{r}}{N_{s}}\right)\right]-\eta^{♢}E\left(\xi P_{t}+P_{c}\right)\right\} \end{array}$$

(16) $$\begin{array}{c} =E\left[\log_{2}\left(1+\frac{P_{r}^{♢}}{N_{s}}\right)\right]-\eta^{♢}E\left(\xi P_{t}^{♢}+P_{c}\right) \end{array}$$

(17) $$\begin{array}{c} <E\left[\log_{2}\left(1+\frac{P_{r}^{♢}}{N_{s}}\right)\right]-\eta^{♡}E\left(\xi P_{t}^{♢}+P_{c}\right) \end{array}$$

(18) $$\begin{array}{c} \leq \max_{P_{t}}\left\{E\left[\log_{2}\left(1+\frac{P_{r}}{N_{s}}\right)\right]-\eta^{♡}E\left(\xi P_{t}+P_{c}\right)\right\}=T\left(\eta^{♡}\right), \end{array}$$

where the first inequality is based on $E(\xi P_{t}+P_{c})>0$. ☐

**Lemma** **2.** *Given $P_{t}^{♣}$ satisfying (a1) and (b) in* (8) *and $\eta^{♣}=\frac{E[\log_{2}\left(1+\frac{P_{r}^{♣}}{N_{s}}\right)]}{E(\xi P_{t}^{♣}+P_{c})}$, we have $T(\eta^{♣})\geq 0$.*

**Proof.**  (19) $$\begin{array}{c} T\left(\eta^{♣}\right)=\max_{P_{t}}\left\{E\left[\log_{2}\left(1+\frac{P_{r}}{N_{s}}\right)\right]-\eta^{♣}E\left(\xi P_{t}+P_{c}\right)\right\}\geq E\left[\log_{2}\left(1+\frac{P_{r}^{♣}}{N_{s}}\right)\right]-\eta^{♣}E\left(\xi P_{t}^{♣}+P_{c}\right)=0. \end{array}$$

☐

**Theorem** **3.** The iterative variable $\eta_{n+1}=\frac{E[\log_{2}\left(1+\frac{P_{r}^{k}}{N_{s}}\right)]}{E(\xi P_{t}^{k}+P_{c})}$ produces an increasing sequence of η values, which converges to the optimal value $\eta^{*}$.

**Proof.**  First, we prove $\eta_{n+1}>\eta_{n}$ for all *n* with $T(\eta_{n})>0$. Lemma 2 makes $T(\eta_{n})\geq 0$. By definition of $\eta_{k+1}$, we have $E\left[\log_{2}\left(1+\frac{P_{r}^{k}}{N_{s}}\right)\right]=\eta_{n+1}E\left(\xi P_{t}^{k}+P_{c}\right)$, thus $T\left(\eta_{n}\right)=E\left[\log_{2}\left(1+\frac{P_{r}^{k}}{N_{s}}\right)\right]-\eta_{n}E\left(\xi P_{t}^{k}+P_{c}\right)=\left(\eta_{n+1}-\eta_{n}\right)E\left(\xi P_{t}^{k}+P_{c}\right)>0$. Again using $E(\xi P_{t}^{k}+P_{c})>0$, we have $\eta_{n+1}>\eta_{n}$.

Then we prove $\lim_{n\to \infty }\eta_{n}=\eta^{*}$. From theorem in [26], we have $T(\eta^{*})=0$, if $\lim_{n\to \infty }\eta_{n}=\eta^{\circ }\neq \eta^{*}$, we must have $\eta^{\circ }<\eta^{*}$. By constructing a sequence $\eta_{n}^{\circ }$ such that $\lim_{n\to \infty }T\left(\eta_{n}^{\circ }\right)=T\left(\eta^{\circ }\right)=0$, and using Lemma 1, we have

(20) $$\begin{array}{c} 0=T\left(\eta^{\circ }\right)>T\left(\eta^{*}\right)=0, \end{array}$$

which is a contradiction. Hence $\lim_{n\to \infty }T\left(\eta_{n}\right)=T\left(\eta^{*}\right)$. Considering the continuous property of T(·), we have $\lim_{n\to \infty }\eta_{n}=\eta^{*}$. ☐

#### 3.1.2. Peak Transmit Power Constraint

When we adopt PTP constraint for the satellite user, the EE maximization problem can be given as

(21) $$\begin{array}{c} \max_{P_{t}}H\left(P_{t}\right)=\frac{E\left[\log_{2}\left(1+\frac{P_{r}}{N_{s}}\right)\right]}{E\left(\xi P_{t}+P_{c}\right)},\\ s.t.\left\{\begin{array}{c} P_{t}\leq P_{m}\;\;\;\;\left(a2\right)\\ E\left(P_{i}\right)\leq I_{th}\;\;\;\left(b\right) \end{array}\right. \end{array}$$

where $P_{m}$ is the PTP constraint limit. It can be proved that (21) is also a nonlinear concave fractional programming problem. Therefore, based on the Dinkelbach’s method, (21) is equivalent to the following optimization problem

(22) $$T\left(\eta \right)=\max_{P_{t}\in S_{2}}E\left[\log_{2}\left(1+\frac{P_{r}}{N_{s}}\right)\right]-\eta E\left(\xi P_{t}+P_{c}\right),$$

where η is a non-negative parameter and $S_{2}$ denotes the set $S_{2}=\left\{P_{t}|P_{t}\in \left(a2\right)\cap \left(b\right)\right\}$. Similar to (9), Lagrange duality method can also be employed to solve (22). If the Lagrangian multipliers with respect to (b) is μ, we can decompose (22) into multiple parallel subproblems with the identical structure for each fading state, which is shown as

(23) $$\max_{0\leq P_{t}\leq P_{m}}D\left(P_{t}\right)=\log_{2}\left(1+\frac{P_{r}}{N_{s}}\right)-\eta \xi P_{t}-\mu P_{i}.$$

Then, we can address (22) by iteratively solving (23) for all fading states with a given μ and updating μ with the subgradient method. Hence, we can finally obtain the optimal allocated power as shown in Theorem 4.

**Theorem** **4.** *The energy-efficient optimal transmit power with PTP constraint for non-real-time applications is given as*

(24) $$P_{t}^{*}=\min\left({\hat{P}}_{t},P_{m}\right),$$

*where ${\hat{P}}_{t}$ can be calculated as*

(25) $${\hat{P}}_{t}={\left[\frac{1}{\left(\eta \xi +\mu G_{I}h_{I}\right)\ln2}-\frac{N_{s}}{G_{S}h_{S}}\right]}^{+}.$$

Note that we can efficiently solve (22) via Theorem 4 with a fixed η and obtain the optimal EE by updating η with the Dinkelbach’s method. This can be achieved by modifying Algorithm 1, where $P_{t}^{k}$ is calculated by (24) not (14) in each iteration. Moreover, only one Lagrangian multiplier μ need to be updated in the modified algorithm. The details are omitted here for simplicity.

### 3.2. Energy-Efficient Optimal Power Allocation for Real-Time Applications

For real-time applications, which are sensitive to delay, such as voice and video, outage capacity (OC) is more appropriate to be considered as the performance metric, which is defined as the maximum constant rate that can be maintained over fading states with a given outage probability [38]. That is to say, the EE of the satellite user for real-time applications is the ratio of the product of the constant OC and the non-outage probability to the average power consumption. In this section, we propose two energy-efficient optimal power allocation schemes under the AIP constraint, which comply with ATP or PTP constraints, respectively.

#### 3.2.1. Average Transmit Power Constraint

With ATP and AIP constraints, the EE maximization problem for real-time applications can be formulated as

(26) $$\begin{array}{c} \max_{P_{t}}H\left(P_{t}\right)=\frac{R_{th}E\left[1-\chi_{s}\right]}{E\left(\xi P_{t}+P_{c}\right)},\\ s.t.\left\{\begin{array}{c} E\left(P_{t}\right)\leq P_{av}\;\left(a1\right)\\ E\left(P_{i}\right)\leq I_{th}\;\;\;\;\left(b\right) \end{array}\right. \end{array}$$

where $R_{th}$ is the prescribed OC of the satellite user and $\chi_{s}$ is an indicator function for the outage event of the satellite user at each fading state, which is expressed as

(27) $$\chi_{s}=\left\{\begin{array}{c} 1,\left[\log_{2}\left(1+\frac{P_{r}}{N_{s}}\right)\right]<R_{th}\\ 0,\;otherwise \end{array}\right..$$

Note that $\chi_{s}$ is not a concave function with respect to $P_{t}$, thus (26) is not a concave fractional programming problem. However, since the numerator and the denominator of $H\left(P_{t}\right)$ in (26) are continuous and non-negative for any $P_{t}\in S_{1}$, (26) can still be solved with Dinkelbach’s method [26]. Similarly, (26) is equivalent to the optimization problem expressed below

(28) $$T\left(\eta \right)=\max_{P_{t}\in S_{1}}R_{th}E\left[1-\chi_{s}\right]-\eta E\left(\xi P_{t}+P_{c}\right),$$

where η is a non-negative parameter. Using the similar method adopted for (9) and (22), (28) can also be decomposed into multiple parallel subproblems with the same structure for each fading state, where the subproblem for a particular state is given as

(29) $$\max_{P_{t}\geq 0}D\left(P_{t}\right)=-R_{th}\chi_{s}-\eta \xi P_{t}-\tau P_{t}-\mu P_{i}.$$

Then, we can address (28) by iteratively solving (29) for all fading states with fixed τ and μ, and updating τ and μ with subgradient method. Since $\chi_{s}$ is a step function, the corresponding turning point can be calculated as

(30) $$P_{th}=\frac{N_{s}\left(2^{R_{th}}-1\right)}{G_{S}h_{S}},$$

where $P_{th}\geq 0$, which is the minimum transmit power required for the satellite user to guarantee $R_{th}$. It is notable that $\chi_{s}=1$ when $P_{t}<P_{th}$ whereas $\chi_{s}=0$ otherwise. We can conclude that the maximum of $D\left(P_{t}\right)$ is $-R_{th}$ when $P_{t}=0$ or $-\left(\eta \xi +\tau +\mu G_{I}h_{I}\right)P_{th}$ when $P_{t}=P_{th}$. Let $P_{t}^{*}$ denote the optimal transmit power for (28), which depends on the relationship between $-R_{th}$ and $-\left(\eta \xi +\tau +\mu G_{I}h_{I}\right)P_{th}$. Therefore, $P_{t}^{*}$ can be given as in Theorem 5.

**Theorem** **5.** *The energy-efficient optimal transmit power with ATP constraint for real-time applications is given as*

(31) $$P_{t}^{*}=\left\{\begin{array}{c} 0,\;P_{th}>\frac{R_{th}}{\eta \xi +\tau +\mu G_{I}h_{I}}\\ P_{th},P_{th}\leq \frac{R_{th}}{\eta \xi +\tau +\mu G_{I}h_{I}} \end{array}\right..$$

For a particular η, (28) can be efficiently solved via (31). Additionally, we can address (26) by modifying Algorithm 1, where replacing (14) with (31) while calculating $P_{t}^{k}$. For brevity, the details are omitted here due to space limitation.

#### 3.2.2. Peak Transmit Power Constraint

If the PTP and AIP constraints are considered for the satellite user, the EE maximization problem for real-time applications should be formulated as

(32) $$\begin{array}{c} \max_{P_{t}}H\left(P_{t}\right)=\frac{R_{th}E\left[1-\chi_{s}\right]}{E\left(\xi P_{t}+P_{c}\right)},\\ s.t.\left\{\begin{array}{c} P_{t}\leq P_{m}\;\;\left(a2\right)\\ E\left(P_{i}\right)\leq I_{th}\;\;\;\;\left(b\right) \end{array}\right. \end{array}$$

Similar to (26), we can solve (32) by introducing the equivalent parameter optimization problem based on the Dinkelbach’s method, which is given as

(33) $$T\left(\eta \right)=\max_{P_{t}\in S_{2}}R_{th}E\left[1-\chi_{s}\right]-\eta E\left(\xi P_{t}+P_{c}\right).$$

Then, we decompose (33) into multiple parallel subproblems with the same structure for all fading states, which can be represented as

(34) $$\max_{0\leq P_{t}\leq P_{m}}D\left(P_{t}\right)=-R_{th}\chi_{s}-\eta \xi P_{t}-\mu P_{i}.$$

Let $P_{t}^{*}$ denote the optimal transmit power. By addressing (34), we can obtain the following results.

In the case of $P_{th}>P_{m}$, where $P_{th}$ is calculated by (30), since the required minimum transmit power to maintain $R_{th}$ is larger than the maximum available transmit power, the satellite user is always in outage. Therefore, $P_{t}^{*}=0$.

In the case of $P_{th}\leq P_{m}$, the maximum of $D\left(P_{t}\right)$ is $-R_{th}$ when $P_{t}=0$ or $-\left(\eta \xi +\mu G_{I}h_{I}\right)P_{th}$ when $P_{t}=P_{th}$, which is the maximum depends on their relationship. If $P_{th}>R_{th}/\left(\eta \xi +\mu G_{I}h_{I}\right)$, the required transmit power to maintain $R_{th}$ is very large, and the satellite user would stop working to save the power, i.e. $P_{t}^{*}=0$. Otherwise, the satellite user transmits with $P_{t}^{*}=P_{th}$.

Based on the above analysis, the optimal transmit power of the satellite user can be summarized as shown in Theorem 6.

**Theorem** **6.** *The energy-efficient optimal transmit power with PTP constraint for real-time applications is given as*

(35) $$P_{t}^{*}=\left\{\begin{array}{c} \begin{array}{c} 0,\;P_{th}>P_{m}\\ 0,\;\frac{R_{th}}{\eta \xi +\mu G_{I}h_{I}}<P_{th}\leq P_{m} \end{array}\\ P_{th},\;P_{th}\leq P_{m},P_{th}\leq \frac{R_{th}}{\eta \xi +\mu G_{I}h_{I}} \end{array}\right..$$

Similarly, we can modify Algorithm 1 to solve (32), where $P_{t}^{k}$ is calculated by (35) in each iteration and only one Lagrangian multiplier μ need to be updated. The details are not given here for simplicity.

## 4. Simulation Results and Analysis

In this section, we present numerical results to evaluate the performance of the proposed energy-efficient optimal power allocation schemes in integrated wireless sensor and cognitive satellite terrestrial networks. In the simulations, we consider the simulation parameters as shown in Table 1 unless otherwise stated [5,15,37]. Besides, the Average Shadowing (AS) scenario is assumed for satellite link [34]. Furthermore, all the simulation results are obtained through Monte Carlo simulations for Shadowed-Rician fading channel and Nakagami-*m* fading channel, which employ $5\times 10^{3}$ realizations.

### 4.1. Non-Real-Time Applications

**Figure 2:**
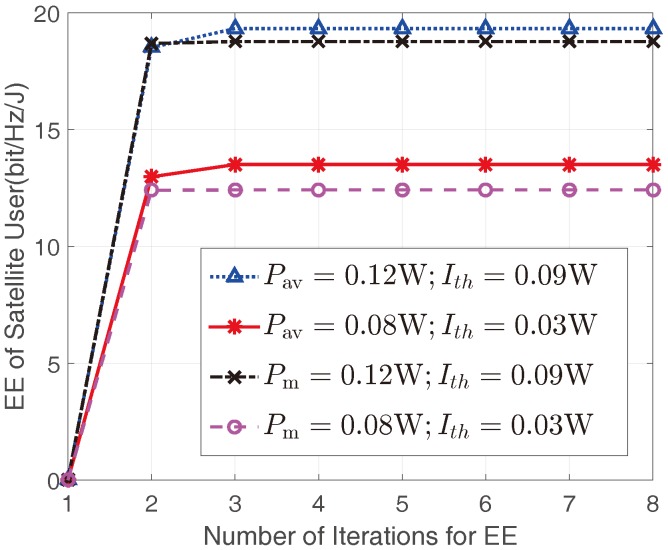
The EEs of the satellite user versus number of the iterations in Algorithm 1 with $m_{I}\;=\;1$ and $\Omega_{I}\;=\;1$.

Figure 2 depicts the EEs of the satellite user versus the number of iterations in Algorithm 1 with different $P_{av}$/$P_{m}$ and $I_{th}$. It can be seen that Algorithm 1 is convergent for all parameters considered, which proves the effectiveness of the proposed iterative algorithm. Moreover, we can find that all the simulation results would converge within 3 iterations. That is to say, the proposed Algorithm 1 can efficiently find the optimal EE for the satellite user.

**Figure 3:**
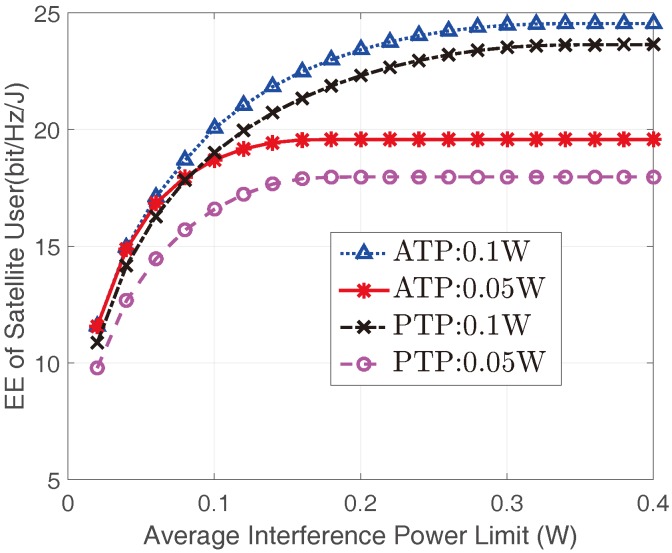
The EEs of the satellite user versus $I_{th}$ with different $P_{av}$ and $P_{m}$ with $m_{I}\;=\;1$ and $\Omega_{I}\;=\;1$.

Figure 3 shows the optimal EEs of the satellite user versus $I_{th}$ with different $P_{av}$ and $P_{m}$ for the non-real-time applications. It can be found that the EEs of satellite user improves with the increase of $I_{th}$. This is because the larger $I_{th}$ is, the more transmit power satellite user can obtain, which correspondingly lead to a higher EE. However, when $I_{th}$ is sufficiently large, the EE of the satellite user would get saturated since the transmit power constraints become the dominant constraints in this case. In addition, our findings suggest that the EEs of the satellite user with ATP constraint are higher than those with the PTP constraint, this is due to the fact that in PTP cases, the satellite user utilizes the instantaneous CSI, which results in a stricter power constraint than those of ATP cases with statistical CSI. Meanwhile, the EEs of the satellite user also improve with the increase of transmit power constraints.

**Figure 4:**
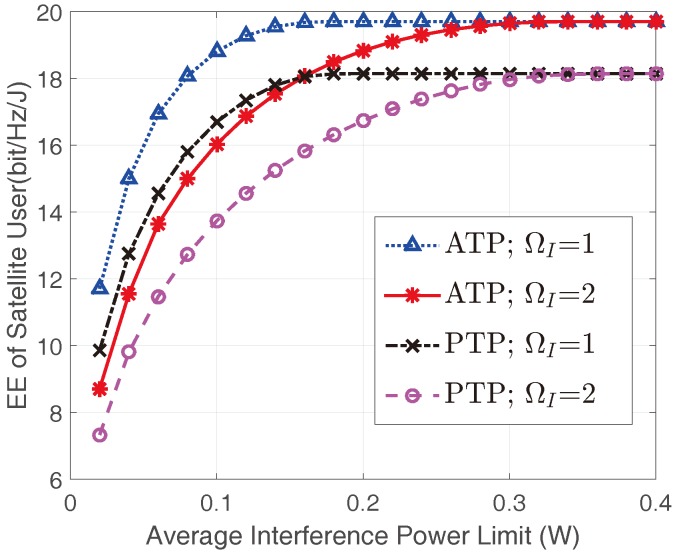
The EEs of the satellite user versus $I_{th}$ with different transmit constraints and interference link conditions with $P_{av}\;=\;P_{m}\;=\;0.05\;W$ and $m_{I}\;=\;1$.

Figure 4 shows the optimal EEs of the satellite user in different terrestrial interference links. All the EEs of the satellite user with ATP constraint are higher than those with the PTP constraint under the same channel conditions, which is consistent with the findings in Figure 3. With the same transmit power constraint, the EE of the satellite user decreases with the increase of $\Omega_{I}$, which can be explained by the fact that the terrestrial interference link would become stronger with larger $\Omega_{I}$. However, the saturated EE values under the same transmit power constraint are identical when $I_{th}$ is large enough, since the transmit power constraints dominate in this case and the limits are the same as mentioned above.

### 4.2. Real-Time Applications

**Figure 5:**
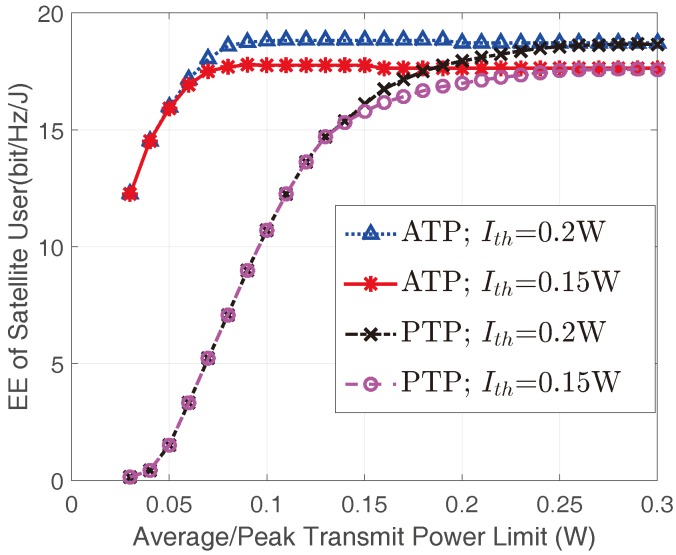
The EEs of the satellite user versus transmit constraint limits for different $I_{th}$ with $m_{I}\;=\;1$ and $\Omega_{I}\;=\;1$.

Figure 5 illustrates the optimal EEs of the satellite user versus $P_{av}$/$P_{m}$ for different $I_{th}$. When $I_{th}$ is relatively small, the obtained EEs under the same transmit power constraint are equal. The reason is that the AIP constraint is inactive while ATP/PTP constraints are tight enough. With the increase of $P_{av}$/$P_{m}$, AIP would be active and larger $I_{th}$ corresponds to higher EEs. Interestingly, for the same $I_{th}$, the EEs for both ATP and PTP constraints converge to the same value. This phenomenon indicates that when $P_{av}$/$P_{m}$ is large enough, the transmit power would be dominated merely by the AIP constraint.

**Figure 6:**
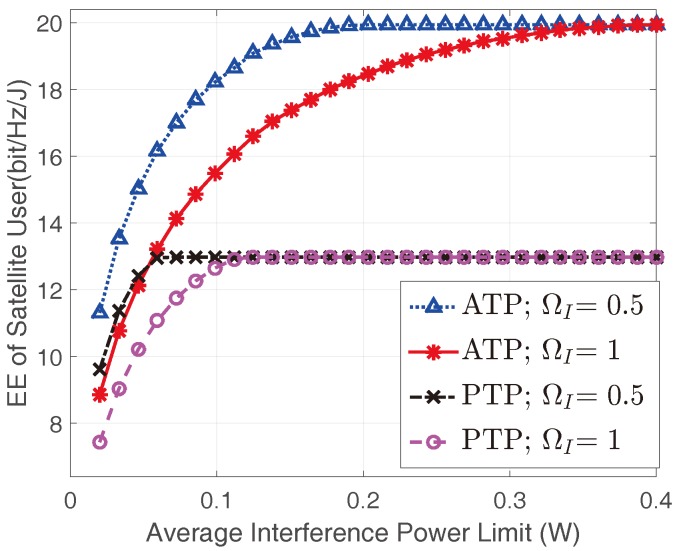
The EEs of the satellite user versus $I_{th}$ for different interference link conditions with $P_{av}\;=\;P_{m}\;=\;0.12\;W$ and $m_{I}\;=\;1$.

Figure 6 shows the optimal EEs of the satellite user versus $I_{th}$ for different $\Omega_{I}$ of terrestrial interference link. Similarly, in the same interference link scenario, the achievable EE under ATP constraint is higher than that of PTP. Furthermore, it is notable that with the increase of $\Omega_{I}$, the EE decrease correspondingly, which means that strong interference link fading is favorable to improve the EE of the satellite user. Finally, we can find the interesting phenomenon that when $I_{th}$ is large enough, the EE of the satellite user would get the same saturated values whatever transmit power constraint is adopted. This is because the AIP is inactive in this situation, and the fading of the interference link has no impact on the EE of the satellite user.

## 5. Conclusions

In this paper, a novel satellite-based WSN is first proposed, which integrates the WSN with the cognitive satellite terrestrial network. Then, the energy-efficient optimal power allocation schemes in cognitive satellite terrestrial networks are proposed for non-real-time and real-time applications, respectively. For both scenarios, AIP constraint is adopted to guarantee the interference power at the primary terrestrial user under a tolerable limit, while ATP and PTP constraints are employed for the transmit power constraint of the satellite user, respectively. In this context, the energy-efficient optimal power allocation problem can be formulated as a nonlinear fractional programming problem, which is solved by combining the Dinkelbach’s method and the Lagrange duality method. Extensive numerical results evaluate the impact of interference power limit, transmit power limits and the interference link quality on the EE of the satellite user. It can be observed that in the same scenario, the optimal EE of the satellite user under ATP constraint is larger than that under PTP constraint. In addition, strong interference link fading is favorable to the performance of the satellite user.

## Figures and Tables

**Table 1:** Simulation Parameters.

Parameters	Values
signal frequency (*f*)	2 GHz
$G_{t,\max}$	42.1 dB
$G_{r,\max}$	52.1 dB
$G_{BS}$	0 dB
θ	$20^{\circ }$
ψ	$50^{\circ }$
$N_{s}$	0.01 W
ξ	0.2
$P_{c}$	0.05 W
$R_{th}$	2 bit/s/Hz
satellite link distance ($d_{s}$)	35,786 km
interference link distance ($d_{p}$)	10 km
$t_{1}$, $t_{2}$	0.1
$\xi_{0}$, $\xi_{1}$, $\xi_{2}$	$5\times 10^{-3}$
$m_{S}$	10
$\Omega_{S}$	0.835
$b_{S}$	0.126
